# Spermidine, but not spermine, is essential for pigment pattern formation in zebrafish

**DOI:** 10.1242/bio.018721

**Published:** 2016-05-23

**Authors:** Hans Georg Frohnhöfer, Silke Geiger-Rudolph, Martin Pattky, Martin Meixner, Carolin Huhn, Hans-Martin Maischein, Robert Geisler, Ines Gehring, Florian Maderspacher, Christiane Nüsslein-Volhard, Uwe Irion

**Affiliations:** 1 Max-Planck-Institut für Entwicklungsbiologie, Abteilung 3, Spemannstrasse 35, Tübingen 72076, Germany; 2 Institut für Physikalische und Theoretische Chemie, Eberhard Karls Universität Tübingen, Auf der Morgenstelle 18, Tübingen 72076, Germany

**Keywords:** Zebrafish, Pigmentation, Pattern formation, Polyamine, Spermidine synthase, Spermine synthase

## Abstract

Polyamines are small poly-cations essential for all cellular life. The main polyamines present in metazoans are putrescine, spermidine and spermine. Their exact functions are still largely unclear; however, they are involved in a wide variety of processes affecting cell growth, proliferation, apoptosis and aging. Here we identify *idefix*, a mutation in the zebrafish gene encoding the enzyme spermidine synthase, leading to a severe reduction in spermidine levels as shown by capillary electrophoresis-mass spectrometry. We show that spermidine, but not spermine, is essential for early development, organogenesis and colour pattern formation. Whereas in other vertebrates spermidine deficiency leads to very early embryonic lethality, maternally provided spermidine synthase in zebrafish is sufficient to rescue the early developmental defects. This allows us to uncouple them from events occurring later during colour patterning. Factors involved in the cellular interactions essential for colour patterning, likely targets for spermidine, are the gap junction components Cx41.8, Cx39.4, and Kir7.1, an inwardly rectifying potassium channel, all known to be regulated by polyamines. Thus, zebrafish provide a vertebrate model to study the *in vivo* effects of polyamines.

## Abstract (precis)

**Summary:** We show that the polyamine spermidine, but not spermine, in addition to more general functions during early development, also specifically regulates colour pattern formation in adult zebrafish.

## INTRODUCTION

One of the most prominent features of adult zebrafish is their pigmentation; the fish display a stereotypical pattern of horizontal dark and light stripes on their flanks and in the anal and caudal fins. Three different types of pigment cells (chromatophores) are responsible for this pattern: melanophores, containing the dark pigment melanin; xanthophores, containing orange pteridine pigments; and iridophores, which contain reflecting guanine platelets (Hirata et al., 2003; Singh and Nüsslein-Volhard, 2015; Irion et al., 2016). All three types of chromatophores are required to establish the pattern. Mutants missing one chromatophore type, *nacre* (*nac*) (Lister et al., 1999), *pfeffer* (*pfe*) (Parichy et al., 2000) or *shady* (*shd*) (Lopes et al., 2008), where melanophores, xanthophores or iridophores are absent, respectively, only produce a rudimentary pattern (Frohnhöfer et al., 2013). In several other mutants the pattern of the adult fish is affected, but all three types of chromatophores are present in nearly normal numbers. In most of these mutants, e.g. *leopard* (*leo, Cx41.8*) (Watanabe et al., 2006), *luchs* (*luc, Cx39.4*) (Irion et al., 2014a), *schachbrett* (*sbr, Tjp1a*) (Fadeev et al., 2015) and *seurat* (*igsf11*) (Eom et al., 2012), the dark stripes are broken up into spots; in a few others, *obelix* (*obe*, also known as *jaguar, Kir7.1*) (Iwashita et al., 2006; Maderspacher and Nüsslein-Volhard, 2003) and *asterix* (*ase*) (Haffter et al., 1996), the width of the stripes is affected. In *leo* and *luc* the affected genes code for two different connexins, the subunits of gap junctions. Gap junctions allow the molecular and electrical coupling between neighbouring cells via the transfer of small molecules and ions (Kumar and Gilula, 1996). Both *leo* and *luc* are required in melanophores and xanthophores (Irion et al., 2014a; Maderspacher and Nüsslein-Volhard, 2003) and it has been suggested that they form heteromeric gap junctions allowing cell-cell communications necessary for normal pattern formation. In *obe*, which is only required in melanophores (Maderspacher and Nüsslein-Volhard, 2003), the affected gene codes for a K^+^ inwardly rectifying (Kir) channel, *kcnj13/Kir7.1*. Kir channels play key roles in the maintenance of the resting membrane potential of many cell types and they contribute to the formation of action potentials in excitable cells such as neurons and cardiomyocytes. The properties of both gap junctions and Kir-channels can be regulated by the binding of polyamines to the channel proteins (Musa et al., 2004; Baronas and Kurata, 2014). Recently it was shown that the N-terminus of connexin41.8, which is affected in *leo* mutants, contains a putative polyamine-binding motif, ExxxE, necessary for the correct pigment pattern formation *in vivo* (Watanabe et al., 2012); the same motif is also present in Cx39.4, which is encoded by *luc* (Irion et al., 2014a).

Polyamines, mainly putrescine, spermidine and spermine, are important for viability, proliferation and differentiation in all cells tested (Miller-Fleming et al., 2015; Wallace, 2009). They are derived from ornithine via decarboxylation (putrescine) and the addition of one (spermidine) or two (spermine) aminopropyl groups (Pegg, 2009). However, the exact roles polyamines fulfil in the cellular physiologies are still largely unclear. They carry several positive charges already at neutral pH and are thought to stabilize negatively charged molecules in the cell, such as RNA, DNA or membrane phospholipids. Polyamines are known to affect gene expression by regulating transcription or translation of mRNAs (Igarashi and Kashiwagi, 2015; Miller-Fleming et al., 2015). Reduced polyamine levels have been associated with cellular senescence and the application of spermidine increases the life span in yeast, flies and human cells (Eisenberg et al., 2009). In many cancers the levels of polyamines are elevated (Thomas and Thomas, 2003). One essential and well-understood function of spermidine in all eukaryotes is its requirement as a substrate for the post-translational modification of a specific lysine residue in eIF5A, leading to the unusual amino acid hypusine [N(ε)-(4-amino-2-hydroxybutyl)-lysine]. Both, eIF5A and the enzymes required for hypusination, are essential genes in mouse, underscoring the importance also of spermidine (Nishimura et al., 2012). Polyamines are also involved in the regulation of several classes of ion channels, e.g. K^+^ inward-rectifier channels, ionotropic glutamate receptors and gap junctions. Binding of polyamines is necessary for the rectification properties of these channels, allowing ions to pass through the pore only in one direction (Donevan and Rogawski, 1995; Lu, 2004; Musa and Veenstra, 2003; Williams, 1997).

Here we describe a novel zebrafish mutant, *idefix* (*ide*). We show that *ide* mutants carry a premature stop codon in the gene coding for spermidine synthase, the enzyme responsible for the synthesis of spermidine from putrescine and S-adenosyl-methioninamine. This is the first instance where the loss of spermidine synthase is described in a vertebrate. Surprisingly, the homozygous mutants are viable, but show a maternal-effect lethal phenotype and a very striking aberration from the wild-type stripe pattern. In agreement with a recent report (Mastracci et al., 2015) we show that the maternal depletion of the enzyme leads to defects in the development of the pancreas. In the homozygous mutants the pigmentation pattern displays wider light areas and narrower and fewer dark stripes, which show frequent interruptions. The sharp boundaries between the stripes are not affected. We demonstrate that the phenotype is caused by a lack of spermidine and not spermine, as mutations in the gene coding for spermine synthase cause no visible phenotype. To determine the polyamine levels in early embryos we established a protocol for separation and detection based on capillary electrophoresis coupled to mass spectrometry (CE-MS), which allowed the sensitive and reliable relative quantification of putrescine, spermidine and spermine ratios. We found that, as predicted, putrescine accumulates in *ide* mutants and the levels of spermidine are greatly reduced. A loss-of-function mutation in the gene coding for spermine synthase leads to very low spermine levels (below the detection limit of the method), however, the fish are viable and fertile and show no visible phenotype. This demonstrates that *in vivo* spermidine has an essential function, whereas spermine does not.

## RESULTS

### *idefix* mutants show pigment pattern irregularities

**Fig. 1.**
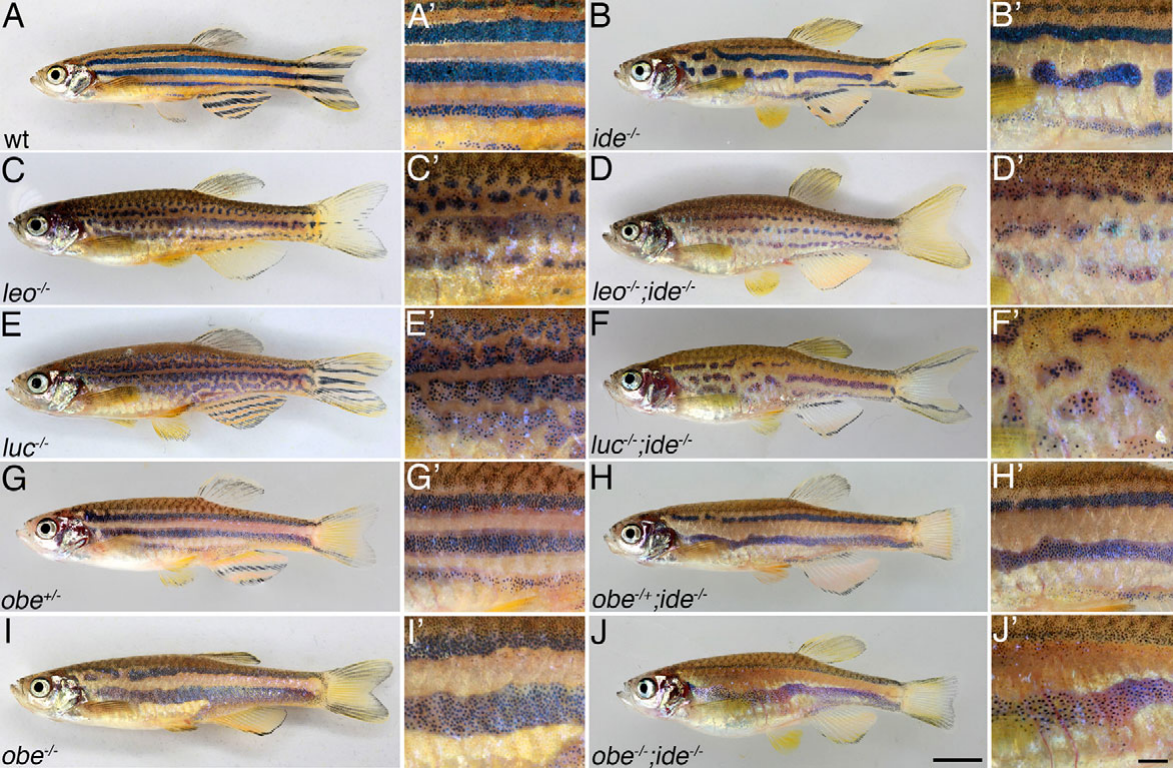
***idefix* mutants show defects in pigment pattern formation.** Wild-type (A,A′) zebrafish show a stereotypic pattern of horizontal dark and light stripes on their flanks and on the anal and caudal fins. In *ide* mutants (B,B′) the light stripe areas are expanded, there are fewer and less regular dark stripes, which frequently show interruptions. The striped pattern in the anal and caudal fins is also disrupted in *ide* mutants. Double mutants of *ide* with *leo* (C-D′), *luc* (E-F′) or *obe* heterozygous (G-H′) and homozygous (I-J′) show a superimposition of both phenotypes. The light stripe areas are expanded in all cases. Scale bars: 5 mm J, 1 mm in J′.

During an ENU-mutagenesis and screen for mutations causing developmental defects in zebrafish larvae we serendipitously identified one recessive mutant where the stripe width of the adult fish is altered. We named this mutant *idefix* (*ide*), based on its superficial resemblance to two other mutants with defects in stripe width, *obelix* (*obe*, also known as *jaguar*) and *asterix* (*ase*). Homozygous *ide* mutants have fewer dark stripes, only 2-3 compared to 4-5 in wild type, in addition these stripes are less regular, usually narrower and often interrupted. The stripes in the anal and tail fins are also affected, they are only partially present in *ide* mutants (Fig. 1A,B). In double mutants, where *ide* is combined with *leo*, *luc* or *obe*, a superimposition of both phenotypes is visible. The light stripe areas are expanded between the spots or broadened dark stripes characteristic for the single mutants (Fig. 1C-J). These findings show that *ide* does not act exclusively through one of the pathways defined by *obe* or *leo* and *luc*; otherwise the phenotype of the loss-of-function mutants would not be altered in the double mutants.

**Fig. 2.**
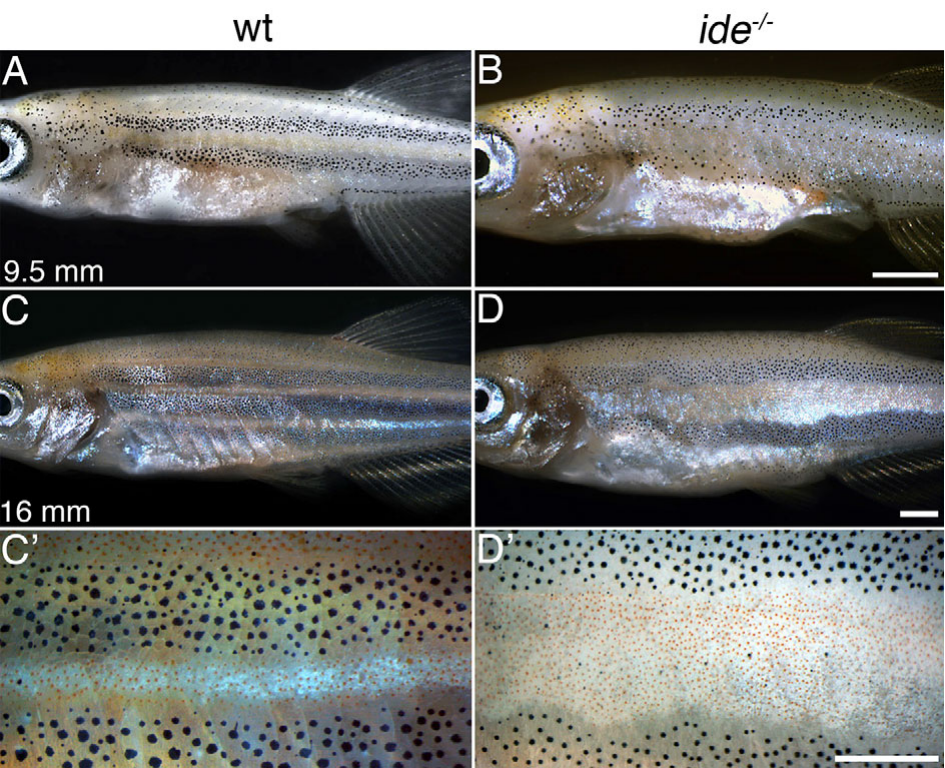
**The *idefix* phenotype first becomes visible during metamorphosis.** (A,B) Wild-type and *ide* mutant zebrafish at stage SP, 9.5 mm standard length. In the mutants the boundaries between the first light stripe and the developing dark stripes are less regular than in wild type. (C-D′) At stage J++, 16 mm standard length, the *ide* phenotype is fully visible. The light stripe area is wider than in wild type and only two dark stripes develop. The xanthophore densities in the light stripes are similar in wild type and ide mutants (C′,D′). Scale bars: 1 mm.

The *ide* phenotype develops during metamorphosis, the early larval pattern is not altered in the mutants. The mutant phenotype is clearly visible at stage SP (9.5 mm SL), with the first light stripe being noticeably wider than in wild type (Fig. 2A-D). The density of xanthophores in the light stripes is not altered in *ide* mutants.

### *idefix* does not act autonomously in pigment cells

**Fig. 3.**
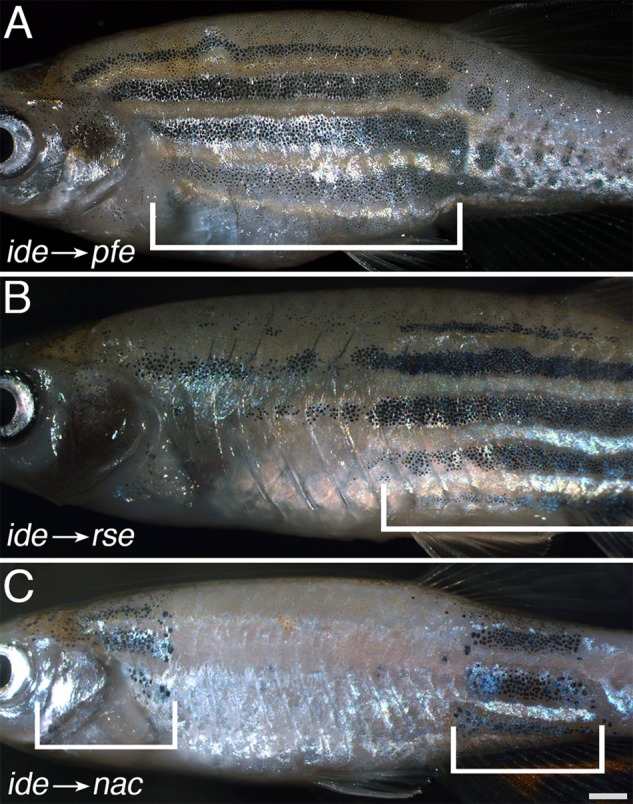
***idefix* mutant chromatophores contribute to a wild-type pattern in chimeric animals.** Chimeric animals derived from blastomere transplantations of *ide* mutant cells into (A) *pfeffer* (*n*=8), (B) *rose* (*n*=6) and (C) *nacre* (*n*=4) hosts. In all three cases the normally striped wild-type pattern is restored in the regions of donor-derived xanthophores (A), iridophores (B) or melanophores (C), indicated by the brackets. Scale bar: 1 mm.

To test whether the function of *ide* is autonomously required in pigment cells we created chimeric animals by transplanting blastomeres from homozygous *ide* mutant donor embryos into *pfeffer*, *rose* or *nacre* hosts, which specifically lack xanthophores, iridophores or melanophores, respectively, and therefore display only a residual, abnormal stripe pattern. In all three cases we found that the *ide* mutant chromatophores can restore the wild-type pattern in the chimeric animals and generate stripes of normal width (Fig. 3). This demonstrates that the gene function of *ide* is not required in pigment cells, but rather influences their behaviour indirectly.

### *idefix* is a loss-of-function allele of *spermidine synthase* (*srm*)

**Fig. 4.**
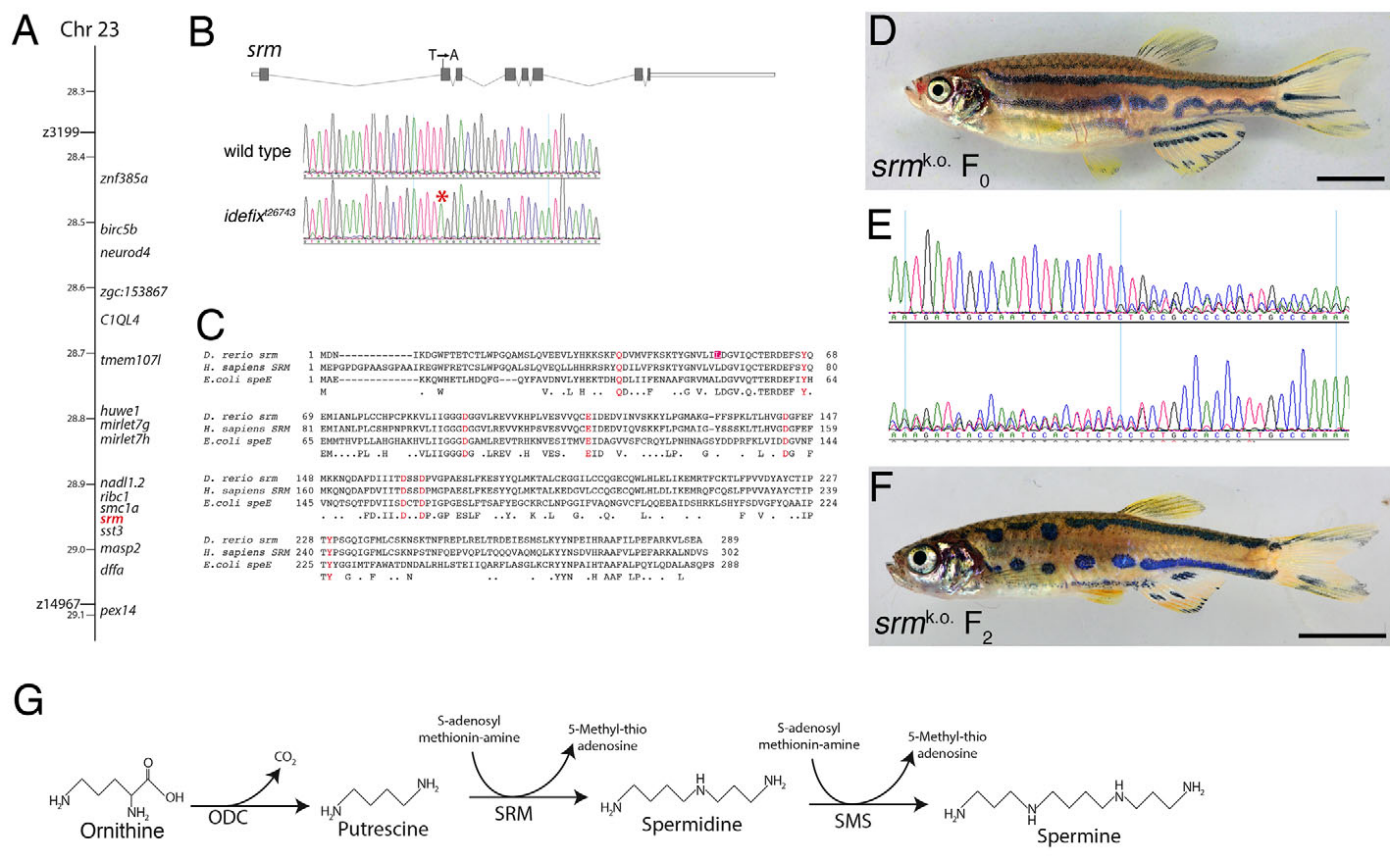
**A premature stop codon in *spermidine synthase* is responsible for the *idefix* phenotype.** (A) The *ide* mutation, *t26743*, was placed between the two z-markers z3199 and z14967 by mapping of meiotic recombinants. The region contains 14 annotated protein coding genes and two microRNA genes. (B) *ide^t26743^*mutants carry a mutation in exon 2 of *srm*. The gene structure is shown in the top panel, the bottom panel shows the sequence chromatograms of wild-type and homozygous mutant fish. The mutated nucleotide is marked with an asterisk. (C) An alignment of the amino acid sequences of spermidine synthases from zebrafish (*Danio rerio*), humans (*Homo sapiens*) and *E.coli*. Positions of conserved amino acid residues important for substrate binding and catalytic activity are highlighted in red. The position of the mutation in *ide^t26743^* (Leu53 to Stop) is underlaid in red. (D) An F_0_ fish is shown, which was obtained after CRISPR-mediated knockout of *srm*. (E) The sequence chromatograms from the fish in D. The induced mutations lead to a deterioration of the signal quality in both directions at the same position. (F) A homozygous mutant F_2_ fish carries an 80 bp deletion and shows the typical *ide* mutant phenotype. (G) The biosynthetic pathway for the generation of the polyamines putrescine, spermidine and spermine from ornithine and S-adenosyl-methionin-amine. Scale bars: 5 mm.

We mapped the mutation, *ide^t26743^,* by meiotic recombination to a region of approx. 630 kb on chromosome 23. This region contains 14 annotated protein-coding genes (Zv8) and two miRNA genes (Fig. 4A). Sequencing of all predicted coding sequences in the entire region and of both miRNA genes revealed only one significant difference between wild-type fish and *ide* mutants, a T to A transversion in the second exon of the gene coding for the enzyme spermidine synthase (*srm*). This mutation leads to a premature termination codon (TTG to TAG) in the resulting mRNA, and is predicted to give rise to a truncated protein of only 52 amino acids (Leu53 to Stop) instead of 289 residues (Fig. 4B).

Spermidine synthase is an enzyme required for polyamine biosynthesis, it catalyses the transfer of an aminopropyl group from decarboxylated S-adenosyl-methionine (S-adenosyl-methioninamine) to putrescine to form spermidine (Fig. 4G). The predicted zebrafish protein shows a very high similarity to spermidine synthases from other organisms, with 87% similarity to the human protein and 56% similarity to the protein from *E.coli*. The amino acid residues that are known to be required for binding to putrescine and S-adenosyl-methioninamine as well as an important catalytic Asp residue are all conserved (Fig. 4C).

Spermidine is a substrate for the synthesis of spermine by addition of another aminopropyl group, a reaction catalyzed by the enzyme spermine synthase (Fig. 4G), which shows some similarity to spermidine synthase.

To confirm that the mutation in *srm* is indeed responsible for the *ide* phenotype, we used the CRISPR/Cas9 system to generate additional loss-of-function alleles in the gene. We used a sgRNA that targets the second exon of the gene and found a very high incidence of mutations in the injected F_0_ larvae. When grown to adulthood almost all injected individuals showed pigment patterning defects very similar to *ide* mutants (Fig. 4D,E). In the F_2_ generation of the CRISPR-injected fish we found animals homozygous for an 80 bp deletion, which display a phenotype identical to the original *ide^t26743^* fish (Fig. 4F). This confirms our mapping and proves that *srm* is the gene affected in *ide* mutants. We found a very high mortality rate in F_1_ larvae from CRISPR-injected F_0_ females, indicating that the maternal-effect lethality seen in *ide* mutants (see below) is likely also due to mutations in *srm*.

### Maternal loss of spermidine synthase leads to lethality and defects in pancreas development

**Fig. 5.**
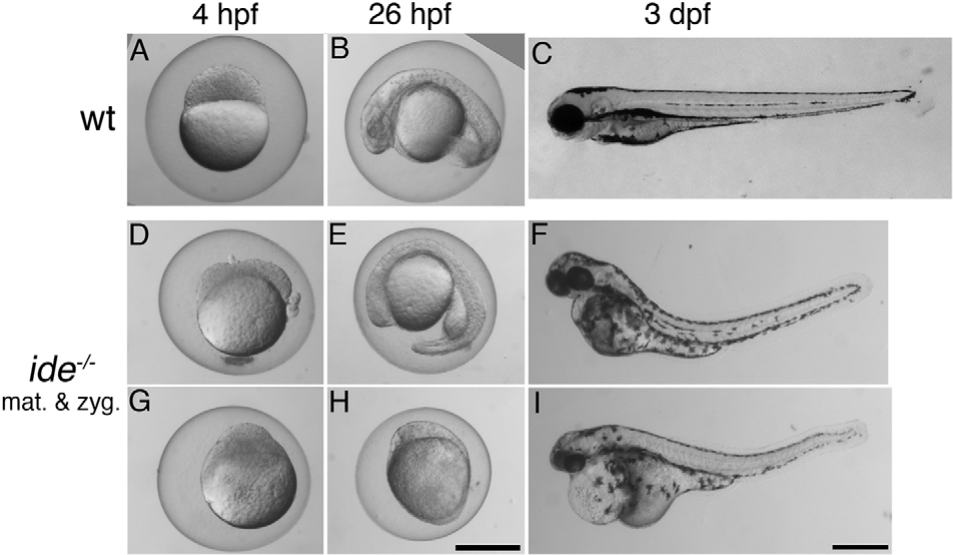
***idefix* mutants show a maternal-effect lethal phenotype.** Wild-type embryos and larvae (A-C) and maternal-zygotic *idefix* mutants (D-I, *ide^−/−^* mat. & zyg.) derived from an incross of two homozygous fish are shown at 4 h post fertilization (hpf), 26 hpf and 3 days post fertilization (dpf). A variable proportion of the mutants show morphological defects, which become apparent at different developmental stages; examples were selected to illustrate the most severe phenotypes. Scale bars: 0.5 mm.

In addition to pigment patterning defects, *ide* mutants show a maternal-effect lethal phenotype. Homozygous mutant larvae derived from homozygous mothers (maternal-zygotic mutants) never survive beyond day 7. They display a large range of different phenotypes, with morphological defects becoming visible in a varying number of embryos during different stages of development. The earliest defects are apparent already during the first few hours of development and some embryos do not complete epiboly and gastrulation. However, often many of them look normal initially and only show defects during the next few days of development, e.g. patterning defects and malformations of the head, heart oedema or curved bodies (Fig. 5A-I). None of the larvae inflate the swim bladder and they do not start feeding.

Heterozygous embryos derived from homozygous mutant females crossed to wild-type males (maternal only mutants) show a similar range of defects. However, sometimes we find zygotic rescue of the maternal-effect lethality; occasionally individual homozygous females can give rise to a small number of viable heterozygous offspring. In summary, the loss of *spermidine synthase* leads to a very variable maternal-effect lethal phenotype that is only rarely rescued by a zygotically provided functional copy of the gene.

**Fig. 6.**
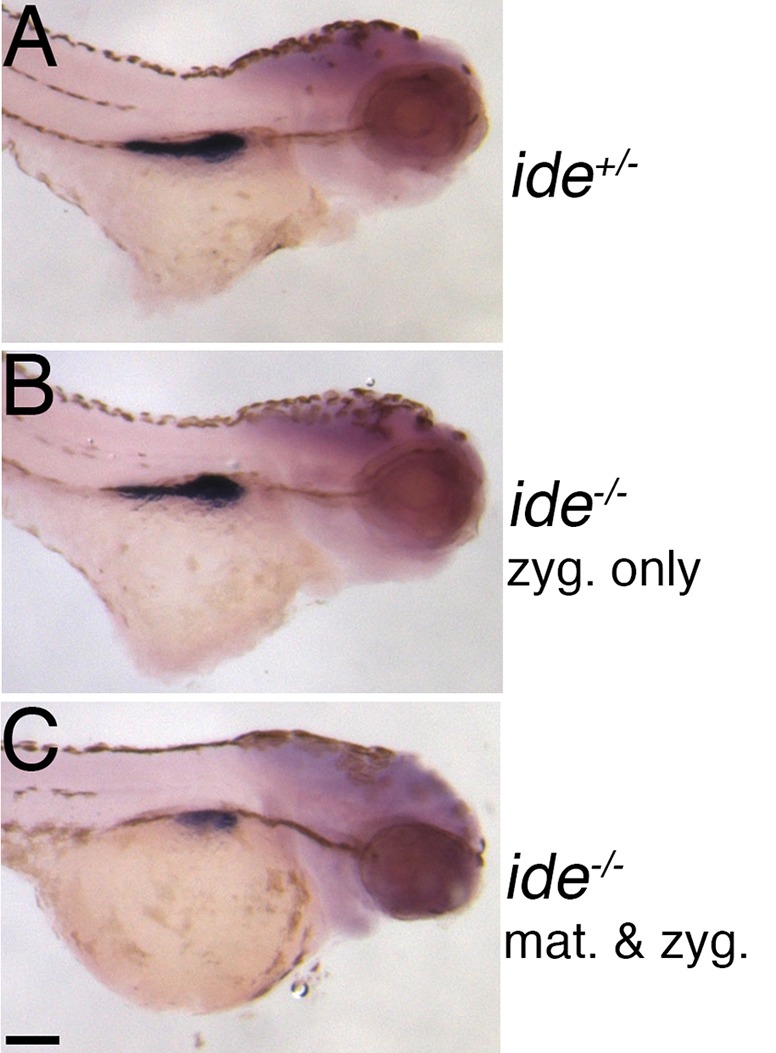
**Development of the exocrine pancreas is defective in *ide* mutants.** Expression of *trypsin* in 72 hpf embryos visualized by *in situ* hybridization. (A) heterozygous (*ide^+/−^*) and (B) homozygous (*ide^−/−^* zyg. only, maternal contribution present) mutants derived from heterozygous parents show no defects (*n*=40). Whereas (C) homozygous mutants derived from an incross of homozygous fish, i.e. zygotic mutants with no maternal contribution (*ide^−/−^* mat. & zyg.), show a severe reduction in the expression domain, indicating defects in the development of the exocrine pancreas (*n*=24). Scale bar: 0.1 mm.

Recently a critical role for polyamine biosynthesis, especially spermidine, in the growth and differentiation of the pancreas in zebrafish was reported (Mastracci et al., 2015). To investigate the effects of the *ide* mutation on the development of the exocrine pancreas we used *in situ* hybridization and assessed the expression of the trypsin gene (*try*), which is specifically expressed in differentiated exocrine cells of the pancreas. We found that homozygous *ide* embryos derived from homozygous parents (maternal-zygotic mutants) showed a very strong reduction in the *trypsin*-expressing region, indicating a severe reduction in the size of the exocrine pancreas (Fig. 6). In contrast, zygotic mutant embryos derived from crosses of heterozygous females with homozygous males, showed no difference in *try* expression compared to their heterozygous siblings. This demonstrates that the eggs are maternally provided with *spermidine synthase* mRNA or the enzyme itself, and that the enzymatic function is critical for embryonic development.

### Loss of spermidine and not spermine causes the *idefix* phenotype

**Fig. 7.**
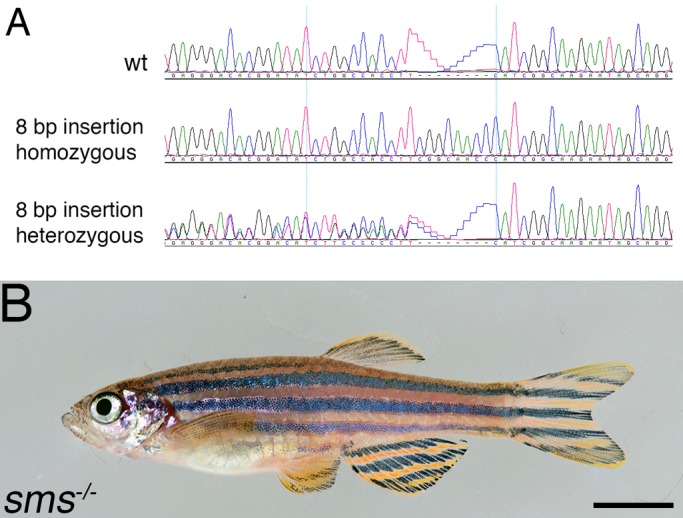
**A knockout mutation in *spermine synthase* has no phenotypic consequences.** (A) Chromatograms of wild-type, homozygous and heterozygous mutants showing that the CRISPR knockout of *sms* resulted in an 8 bp insertion. (B) Homozygous fish are viable and fertile and show no phenotype. Scale bar: 5 mm.

Spermidine is the substrate for the synthesis of the longer polyamine spermine. To investigate whether the *ide* phenotype is directly caused by the lack of spermidine or whether the absence of spermine is responsible, we generated knock out mutants for the gene encoding spermine synthase (*sms*). We used the CRISPR/Cas9 system to target exon 2 of the gene and recovered a mutant with an 8 bp insertion, leading to a frame shift resulting in a truncated protein of 50 amino acids followed by 20 unrelated residues and then a stop codon. Fish homozygous for this mutation are viable, fertile and normally pigmented (Fig. 7). This shows that it is the absence of spermidine that leads to the *ide* phenotype; spermine is not essential for zebrafish.

### Quantification of polyamine levels in zebrafish embryos

**Fig. 8.**
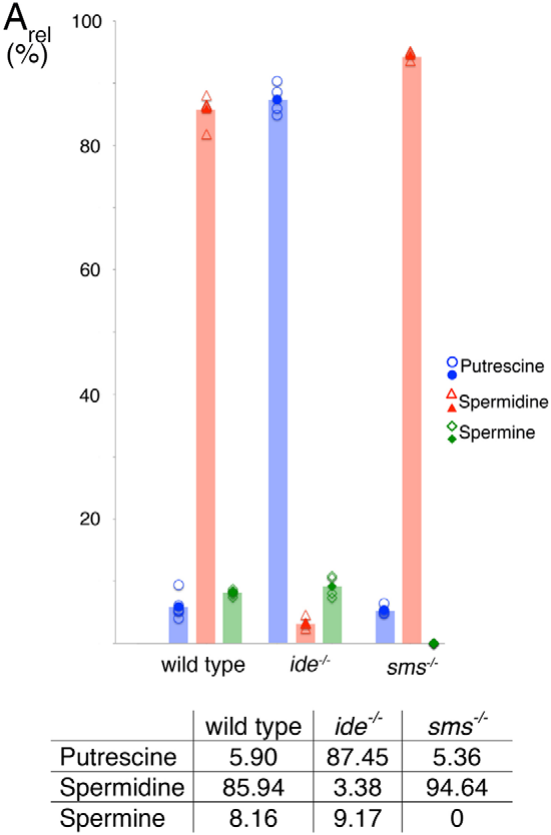
**Polyamine ratios in wild-type and mutant embryos.** The peak area ratios (A_rel_) of the three polyamines measured in wild-type (*n*=6), *idefix* (*ide^−/−^*, *n*=4) and *spermine synthase* (*sms^−/−^*, *n*=4) mutant embryos are shown as open symbols. Peak area ratios were calculated as A_x,rel_=A_x_/(A_putrescine_+A_spermidine_+A_spermine_), x=the respective polyamine. Peak area ratios were normalized to 100% for the sum of the signals in each sample to improve comparability. The mean values shown in the table are depicted as filled symbols and bars. Ionization efficiencies are: putrescine<spermine∼spermidine.

To corroborate the above findings for the roles of polyamines, especially spermidine, during embryonic development and in pigment patterning we quantified the relative abundances (based on peak areas) of putrescine, spermidine and spermine in embryos. Therefore we established an analytical method based on CE-MS. Polyamine separation by CE-MS features both advantageous as well as challenging aspects compared to classical chromatographic approaches. For the analysis of fish egg samples of spermine and spermidine synthase mutants, only limited sample amounts were available, though using CE-MS allowed us to measure repetitively from sample volumes of only 15-20 µl. Furthermore, the simple extraction protocol with just hydrochloric acid and a subsequent precipitation of proteins with 70% acetonitrile, which was compatible with CE-MS analysis, proved to be straight-forward, reducing potential analyte loss during sample pretreatment to a minimum. Neither polyamine derivatization, which is commonly conducted when HPLC is used for polyamine analysis (Magnes et al., 2014); nor sample filtration (Simo et al., 2008), salt removal by e.g. solid phase extraction or other further purification steps (Lange et al., 2002) were required. We determined the ratios of the three polyamines in early embryos 4 hpf (hours post fertilization). Peak areas for each amine were determined. Fig. 8 shows the percentage for the peak area of each amine relative to the sum of all three peak areas (ionization efficiencies: putrescine<spermine∼spermidine). In the *sms* mutants, the spermine content was below the detection limit while the ratio between spermidine and putrescine remained unchanged compared to the wild type samples. In *ide* mutant embryos however, we found that the putrescine content was significantly increased due to the deactivation of spermidine synthase and accumulation of the enzyme substrate. Notably, low quantities of both spermine and spermidine are still detectable, indicating uptake of at least spermidine e.g. via food or from the water.

## DISCUSSION

Here we describe *idefix*, a zebrafish mutant with specific defects in the formation of the adult pigment pattern. We identify the underlying mutation, a premature stop codon in the gene coding for spermidine synthase (*srm*), which is predicted to lead to a complete loss of enzymatic function. Using BLAST (Altschul et al., 1990) searches we could not detect any other gene in the zebrafish genome coding for a similar protein that might potentially fulfil the same enzymatic function; indeed, the most similar sequence identified is the gene coding for spermine synthase. Therefore, we conclude that zebrafish unable to synthesize any spermidine are viable. Measurements show that in early embryos derived from homozygous mutant females the levels of spermidine are drastically reduced, while putrescine, the substrate for spermidine synthase, accumulates. The phenotypes we observe in *ide* mutants might be caused by a lack of spermidine or the accumulation of putrescine. We think that for the adult pigment patterning defects clearly the lack of spermidine is responsible, because the *ide* phenotype is very similar to the phenotype described for zebrafish overexpressing the catabolic enzyme spermine spermidine acetyl transferase (Watanabe et al., 2012). For the early embryonic phenotype the situation is less clear and could possibly result from a combination of putrescine toxicity and a lack of spermidine, which is required for many cellular processes and clearly involved in pancreas development (Mastracci et al., 2015). Whereas spermidine synthase or spermidine has to be supplied maternally to support early development; we assume that the fish larvae and adults are able to take up sufficient amounts of spermidine from the water or their diet. Relatively low levels of spermidine are enough to allow survival of the mutants; however, during pigment pattern formation there is obviously a requirement for higher levels, which cannot be met. It is tempting to speculate that these different requirements for spermidine reflect different physiological processes; e.g. general translational efficiency that requires eIF5A and its hypusination in embryos where cells are very rapidly dividing, as opposed to more specific regulation of cell-cell communication via trans-membrane channels and gap junctions during pigment pattern formation.

The maternal-effect phenotype we observe in *ide* mutants is extremely variable, morphological defects maybe visible already after a few hours of development in a varying proportion of the embryos; or, many of them might develop seemingly normally in the beginning, but die during later stages. It is possible that the nutritional status of the females influences this variability and perhaps also the rate of zygotic rescue, which is generally very low.

To determine the relative ratios of the three different polyamines in early embryos we used CE-MS. The most prominent challenge in polyamine analysis is related to the analytes' extreme charge-to-size ratio and thus their very high effective electrophoretic mobility even at relatively high pH. In fact, the polyamines migrated in between matrix potassium and sodium ions (visible via adduct formation on the internal reference masses). On the one hand, this extreme mobility gives rise to a high selectivity of the method (hardly any other organic ions can be expected to co-migrate), but on the other hand, a good separation from potassium and sodium ions had to be achieved to ensure reliable quantitative data. Sufficient resolution was reached using long capillaries of 100 cm. Even though migration time was significantly increased compared to 65 cm capillaries (data not shown), analytes were still detected within less than 10 min in neutrally coated capillaries. The analysis of polyamines using bare fused silica capillaries was reported (Simo et al., 2008). However, one has to keep in mind that polyamines have also been used as dynamic coating agents in the background electrolyte in CE analysis to prevent adsorption of analytes, which contradicts the use of bare fused silica capillaries (Eckhardt et al., 2004). In this study, we thus decided to use neutrally AAEE (*N*-acryloylamido-ethoxyethanol)-coated capillaries to ensure stable separation conditions with reduced analyte but also matrix component adsorption. We found a strong accumulation of putrescine combined with very low levels of spermidine in *ide* mutants, suggesting that there is some uptake of spermidine via the food or water. In *sms* mutants spermine levels are below the detection limit, whereas the ratio of putrescine and spermidine are unaltered compared with wild type. These data are in good agreement with the notion that polyamine biosynthesis is mainly regulated through ornithine decarboxylase (ODC) and S-adenosyl-methionine decarboxylase (AdoMetDC), two enzymes that act upstream of spermidine synthase.

The formation of the striped pattern in zebrafish depends on direct cellular interactions between the different types of chromatophores that lead to changes in cell shape and behaviour in iridophores (Singh et al., 2014) and xanthophores (Mahalwar et al., 2014). The channels known to mediate some of these cellular interactions are regulated by polyamines. Through a series of transgenes and rescue experiments it was concluded that the polyamine sensitivity of gap junctions formed by Cx41.8 is required for the formation of the striped pattern in zebrafish (Watanabe et al., 2012). The second connexin, Cx39.4, also has the characteristic ExxxE motif, a predicted polyamine-binding site, in its cytoplasmic N-terminus (Irion et al., 2014a; Watanabe et al., 2015). In addition, rectification of Kir7.1, which is encoded by the *obe* gene, is induced by intracellular binding of Mg^2+^ or polyamines to the channel (Hibino et al., 2010). However, whether the *ide* phenotype is produced via mis-regulation of the heteromeric gap junctions formed by Cx41.8 and Cx39.4, or of the Kir7.1 channel is difficult to say because it is expected that the lack of spermidine leads to a phenotype different from the one caused by loss-of-function of the channels. The phenotypic analysis of *ide* double mutants with *leo*, *luc* or *obe* suggests that *ide* doesn't exclusively affect one of these channels, but possibly both the gap junctions and potassium channels, or even some other as yet unknown channel. Whereas *leo*, *luc* and *obe* only affect melanophores and xanthophores, an important function of iridophores in pigment patterning has been described recently (Frohnhöfer et al., 2013; Patterson and Parichy, 2013). During the formation of the pattern iridophores proliferate and migrate in the skin and change from a dense form present in the light stripes to a lose form in the dark stripe regions (Singh et al., 2014). The *ide* phenotype, with the substantial broadening of the light stripe area in the mutants, could be due to a change in the behaviour of the iridophores. It was also speculated that additional, so far unidentified, connexins might act in iridophores together with Tjp1A (Fadeev et al., 2015). While the two connexins known to function in melanophores and xanthophores contain spermidine-binding motifs, connexins in iridophores might belong to a different class and lead to the formation of rectifying heterotypic gap junctions with the connexins in xanthophores or melanophores. If Cx41.8 is expressed only in melanophores, but not in xanthophores (Watanabe et al., 2012; Watanabe and Kondo, 2012), a phenotype very similar to *ide* is produced. Thus, the loss of Cx41.8 function in xanthophores has similar consequences as the loss of spermidine, arguing for a function of heterotypic polarized gap junctions in the patterning process.

Blastomere transplantations showed that the product of the *ide* gene, the enzyme spermidine synthase, is not required in the chromatophores themselves. We assume that spermidine is produced in other tissues and then imported into the pigment cells to function there in the cytoplasm. This makes *ide* different from other genes, like *bonaparte* or *karneol*, which also affect the formation of the striped pattern indirectly. In the case of *bonaparte*, which codes for basonuclin 2, it was speculated that it is required for the local production of a survival factor chromatophores in the skin (Lang et al., 2009). *karneol*, which codes for endothelin-converting enzyme 2, is necessary for the local processing of endothelin ligands, which in turn influence iridophore development and survival (Krauss et al., 2014). *ide*, on the other hand, is less likely to influence the behaviour of the pigment cells by changes in the local tissue environment, but rather more systemically by providing enough spermidine for the regulation of cell-cell communications.

In contrast to *ide*, there is no obvious phenotype in *spermine synthase* mutants. Fish homozygous for a loss-of-function allele generated with the CRISPR/Cas9 system are viable and fertile and show no defects in their pigment pattern. We could not detect spermine in embryos from these mutants; however, putrescine and spermidine are present in a normal ratio. These findings underscore the notion that *in vivo* spermidine is more important than spermine. Similar results have been found in yeast and higher animals (Hamasaki-Katagiri et al., 1998; Pegg and Michael, 2010). Cellular polyamine levels are regulated at the level of synthesis, degradation or transport. Regulation of synthesis occurs mainly through ornithine decarboxylase (ODC) and S-adenosyl-methionine decarboxylase (AdoMetDC), the two rate-limiting enzymes. So far, mutations in spermidine synthase have not been described in metazoans, presumably due to early lethality. In mouse, the knock out of *Ornithine Decarboxylase* leads to early embryonic lethality (Pendeville et al., 2001); however mutations in spermine synthase have been identified in mouse and humans. Mutations in spermine synthase lead to the Gyro phenotype in mouse (Meyer et al., 1998) and cause Snyder–Robinson syndrome in humans, an X-linked mental retardation condition characterized by intellectual disability, hypotonia, facial asymmetry and unsteady gait (Cason et al., 2003).

The mutation in *spermidine synthase* we describe here provides evidence that spermidine, in addition to a more general role in translational regulation, mediated via eIF5A, is also likely to be directly required *in vivo* for the regulation of cell-cell communications via gap junctions and Kir channels. This process seems to be particularly sensitive to diminished spermidine levels and might provide an opportunity to further investigate polyamine metabolism and transport.

## MATERIALS AND METHODS

### Fish husbandry

Zebrafish were maintained as described earlier (Brand et al., 2002); we used TU as wild type and the following mutants: *idefix^t26743^*, *obelix^txg6^* (Irion et al., 2014a), *asterix^ts212^*, *leo^t1^* (Watanabe et al., 2006). Staging of juveniles was done according to Parichy et al., (2009). For photography fish were anaesthetized in 0.004% MS-222 (Sigma) and imaged with a Canon D5MarkII/Macro 100. For Figs 2 and 3 fish were fixed with 4% formaldehyde/0.08% glutaraldehyd (Sigma) and photographed under a Leica MZ1 stereomicroscope. Images were processed in Adobe Photoshop. All animal experiments were carried out in accordance with the guidelines of the Max-Planck-Society and approved by the Regierungspräsidium Tübingen, Baden-Württemberg, Germany (Aktenzeichen: 35/9185.81-5; 35/9185.46-5 and 35/9185.82-7).

### Transplantations

Chimeric animals were generated by blastomere transplantations as described previously (Kane and Kishimoto, 2002). Donor embryos were homozygous for *ide^t26743^*, hosts were *nacre^w2^* (Lister et al., 1999), *pfeffer^tm236b^* (Haffter et al., 1996) and *rose^tlf802^* (Frohnhöfer et al., 2013).

### Genetic mapping

Genetic linkage was determined as described previously (Geisler et al., 2007). For the sequencing of the coding regions total RNA was extracted from fin clips of homozygous mutant fish using TRIzol Reagent (Invitrogen) according to the manufacturer's protocol. To generate cDNA from this RNA SuperscriptII (Invitrogen) was used. For PCR and sequencing the oligonucleotide primers listed in Table S1 were used.

The microRNA genes (*miR-let-7g*, *miR-let-7h*) were amplified by PCR from genomic DNA and sequenced with the following oligonucleotide primers: 5′-CTGGGATTGGAACAGTCAATGG-3′, 5′-TGAGAGTCAGGATTGGAGTCGG-3′.

### RNA *in situ* hybridization

A 532 bp fragment of the *try* coding sequence was amplified by PCR from cDNA with the following primers: 5′-TGAACAGCGGCTACCACTTC-3′, 5′-TAACCCCAGGACACGATACC-3′, and cloned into pGEM-Teasy (Promega). The resulting plasmid was linearized with SacI and used as template to generate a DIG-labelled probe by *in vitro* transcription with T7 RNA polymerase using the DIG-RNA labelling Mix (Roche). Morphologically normal looking embryos were collected and fixed at 72 hpf, the *in situ* hybridization was carried out according to standard procedures (Thisse and Thisse, 2008). The stained embryos were mounted in glycerol and photographed using a Zeiss Discovery.V20 stereomicroscope. Afterwards they were genotyped using the following oligonucleotide primers: 5′-GTCATCTCGCATGATGCATTCTTC-3′, 5′-TGACCACCTCTCTTAGGACACCAC-3′.

### CRISPR/Cas9 mediated gene knock-outs

Loss-of-function mutations in the genes coding for spermidine synthase (*srm*) and spermine synthase (*sms*) were induced with the CRISPR/Cas9 system as described in (Hwang et al., 2013; Irion et al., 2014b). The following oligonucleotides were used to generate DNA templates in pDR274 for *in vitro* transcription of sgRNAs: *srm*, 5′-TAGGGCAAGGGTGGCAGCAGAG-3′, 5′-AAACCTCTGCTGCCACCCTTGC-3′; *sms*, 5′-TAGGATATCTGGCCACCTTCAT-3′, 5′-AAACATGAAGGTGGCCAGATAT-3′.

For genotyping, fin biopsies were taken and the genomic regions were amplified by PCR and sequenced with the following primer pairs: *srm*, 5′-GTCATCTCGCATGATGCATTCTTC-3′, 5′-TGACCACCTCTCTTAGGACACCAC-3′; *sms*, 5′-TTTGGAAAGGGCTTTTAGGG-3′, 5′-GCCACACACAAAACAGGAAG-3′.

### Determination of polyamine levels by CE-MS

One hundred zebrafish embryos (4 hpf) of each genotype were washed twice with ddH_2_O to remove buffer components, especially sodium and potassium ions, and homogenized with a glass homogenizer and pestle. For polyamine extraction, 50 µl aliquots were incubated with 10 µl of HCl (c=0.6 mol/l) to obtain a final solution with c(HCl)=100 mmol/l for extraction. After 30 min incubation time at room temperature, 140 µl of acetonitrile were added in order to remove matrix components such as proteins via precipitation. After 10 min incubation time and 5 min centrifugation (16,000 ***g***), the supernatant was used for CE-MS analysis without further sample pretreatment.

#### Capillary electrophoresis

An Agilent 7100 capillary electrophoresis system (Agilent Technologies, Waldbronn, Germany) was used for CE analysis. Due to the high mobility of polyamines, a 100 cm capillary coated with polymerized *N*-acryloylamido-ethoxyethanol (inner diameter 50 µm, outer diameter 360 µm) was used for separation. The capillary coating was generated in-house, coating synthesis was adapted from (Chiari et al., 1995). A 3:1 (v/v) mixture of acetic acid:formic acid, each c=1 mol/l, was used as running buffer. Separations were carried out at 30 kV. The extracted fish egg samples were injected hydrodynamically (5 s at 100 mbar). Before each run, the capillary was flushed with running buffer for 500 s to remove potential protein or cell matrix residues.

#### Mass spectrometry

An Agilent 6550 iFunnel Q-ToF-MS (Agilent Technologies, Santa Clara, CA, USA) was used for MS detection. The CE was coupled to the mass spectrometer via a coaxial sheath liquid interface from Agilent. The sheath liquid, a 50:50 (v/v) mixture of water:isopropanol with 0.1% formic acid, was delivered by an 1260 Infinity isocratic pump (Agilent Technologies, Waldbronn, Germany) at a flow rate of 5 µl/min. A Dual Jetstream ion source was used. The nebulizer pressure was set to 0.34 bar, the drying gas flow rate was 11 litres/min at a temperature of 150°C. The sheath gas flow was set to 3.5 litres/min at 195°C. A capillary voltage of 4000 V, a nozzle voltage of 2000 V and a fragmentor voltage of 380 V were used to ensure efficient ion transmission into the MS. The mass range was set to m/z=50–1 700 and the data acquisition rate was 2 spectra/s. The instrument was calibrated using the G1969-85000 Q-TOF standard calibration tune mix from Agilent. To ensure maximum mass accuracy, online mass recalibration was performed.
